# The prognostic role of intragenic copy number breakpoints and identification of novel fusion genes in paediatric high grade glioma

**DOI:** 10.1186/2051-5960-2-23

**Published:** 2014-02-18

**Authors:** Diana Carvalho, Alan Mackay, Lynn Bjerke, Richard G Grundy, Celeste Lopes, Rui M Reis, Chris Jones

**Affiliations:** Divisions of Molecular Pathology and Cancer Therapeutics, Institute of Cancer Research, 15 Cotswold Road, Sutton, Surrey, SM2 5NG UK; University of Coimbra, Palácio dos Grilos, R. da Ilha, Coimbra, 3000-214 Portugal; Life and Health Sciences Research Institute (ICVS), School of Health Sciences, University of Minho, Braga, and ICVS/3B’s-PT Government Associate Laboratory, Braga/Guimarães, Portugal; Childhood Brain Tumour Research Centre, University of Nottingham, Kings Meadow Campus, Lenton Lane, Nottingham, NG7 2NR UK; Molecular Oncology Research Center, Barretos Cancer Hospital, Barretos, SP Brazil

**Keywords:** Fusion, Paediatric, Glioblastoma, Copy number, Intragenic

## Abstract

**Background:**

Paediatric high grade glioma (pHGG) is a distinct biological entity to histologically similar tumours arising in older adults, and has differing copy number profiles and driver genetic alterations. As functionally important intragenic copy number aberrations (iCNA) and fusion genes begin to be identified in adult HGG, the same has not yet been done in the childhood setting. We applied an iCNA algorithm to our previously published dataset of DNA copy number profiling in pHGG with a view to identify novel intragenic breakpoints.

**Results:**

We report a series of 288 iCNA events in pHGG, with the presence of intragenic breakpoints itself a negative prognostic factor. We identified an increased number of iCNA in older children compared to infants, and increased iCNA in *H3F3A* K27M mutant tumours compared to G34R/V and wild-type. We observed numerous gene disruptions by iCNA due to both deletions and amplifications, targeting known HGG-associated genes such as *RB1* and *NF1*, putative tumour suppressors such as *FAF1* and *KIDINS220*, and novel candidates such as *PTPRE* and *KCND2*. We further identified two novel fusion genes in pHGG – *CSGALNACT2:RET* and the complex fusion *DHX57:TMEM178:MAP4K3*. The latter was sequence-validated and appears to be an activating event in pHGG.

**Conclusions:**

These data expand upon our understanding of the genomic events driving these tumours and represent novel targets for therapeutic intervention in these poor prognosis cancers of childhood.

**Electronic supplementary material:**

The online version of this article (doi:10.1186/2051-5960-2-23) contains supplementary material, which is available to authorized users.

## Background

DNA copy number and gene expression studies have highlighted key distinctions between high grade gliomas (HGG) arising in childhood and far more commonly, much later in adult life [1–4]. Indeed, recent exome-level sequencing initiatives have conclusively shown the existence of subgroups of HGG marked by distinct driver mutations [5], which are significantly enriched in young children (*H3F3A* K27M), teenagers and young adults (*H3F3A* G34R/V), and middle-aged adults (*IDH1/2*) [6]. Specific driving events for infants and elderly patients remain to be elucidated, however they too represent biological sub-entities, with infants having few genomic alterations [4], and elderly patients harbouring frequent amplification of *EGFR* and other genomic events [2, 3].

The identification of driving genetic alterations at the DNA copy level are necessarily most commonly focussed on assessing the amplification/deletion of genes in their entirety, and approaches to ascribe significance to genomic events make use of overlapping regions across multiple samples to find genes consistently within regions of gain/loss [7]. This approach has the result of ignoring genes for whom the breakpoint, *i.e.* the specific location of copy number change, is found within the coding regions. Such events may be more than mere bystanders of the “driving” aberration, and may themselves play significant roles in tumour initiation and maintenance.

One key implication of copy number breakpoints occurring within genes is the possibility of generating novel fusions. Gene fusions can occur through both intra- and inter-chromosomal translocations, bringing together coding regions from two or more genes within a single reading frame allowing expression of a novel protein. Such gene fusions are common in cancer, but have historically been thought to be largely restricted to haematological malignancies and selected solid tumours such as sarcomas. Recent evidence has overturned this, with numerous novel gene fusions being discovered in a wide range of cancer types, exemplified by the identification of common *TMPRSS2:ERG* fusions in prostate cancer [8] and the *EML4:ALK* fusion in non-small cell lung cancer [9].

The first fusion gene found in glioblastoma was the rearrangement located at an amplified region at chromosome 4q12, resulting in the fusing of the kinase domain of *PDGFRA* with the regulatory domains of *KDR* (*VEGFR2*) [10]. This *KDR:PDGFRA* was found to be activating and tumorigenic, however to date only a single additional case has been found, in a paediatric high grade glioma (pHGG) [11], and thus these fusions do not represent a common event. Another low frequency fusion has more recently been identified in approximately 3% of adult HGG, involving *FGFR1* or *FGFR3* partnering with *TACC1* or *TACC3*[12]. These *FGFR:TACC* fusions have been shown to localize to mitotic spindle poles, have constitutive kinase activity and induce mitotic and chromosomal segregation defects and aneuploidy [12]. The types of integrated analysis that identified these mutations have also begun to identify more common rearrangements, such as numerous fusions involving *EGFR*, the most frequently seen partner producing the EGFR-SEPT14 fusion demonstrated to activate STAT3 signaling and confer mitogen independence and sensitivity to EGFR inhibition [13].

Such analyses are clearly proving extremely valuable in furthering our understanding of HGG biology and generating novel targets for therapeutic intervention. As similar approaches are yet to be undertaken in the paediatric setting, we have applied an algorithm designed to identify intragenic copy number breakpoints in our previously published study of DNA copy number [4]. We identify numerous potentially functional gene disruptions and a novel validated complex fusion, *DHX57:TMEM178:MAP4K3.*

## Methods

### Published DNA copy number data

We previously carried out a DNA copy number profiling study of 100 pHGG cases on Affymetrix 500 K SNP arrays [4]. The data have been deposited at the Gene Expression Omnibus (GEO, http://www.ncbi.nlm.nih.gov/geo/) with accession number GSE19578. Copy number assignment was carried out as per the original publication, using Affymetrix Genotyping Analysis Software (GTYPE version 4.s) improved using Bayesian Linear Model with Mahalanobis distance classifier algorithm (BRLMM) and standard dChipSNP normalization and model-based expression algorithms [4]. Log_2_-transformed data was used for all subsequent analysis in the present study.

### iCNA algorithm

We implemented the iCNA algorithm developed as part of the GTS package under R2.11.0 (cbio.mskcc.org/~brennan) [14]. Breakpoints are calculated according to user-defined ‘delta’ values representing shifts in log_2_ ratios between two contiguous genomic regions after segmenting the copy number data using circular binary segmentation (cbs) [15]. Using a delta of 0.4, breakpoint boundaries are identified and errors estimated by permutation-based calculations of neighbouring probe data. Confidence intervals are assigned and those falling within the 95% window considered ‘high confidence’. An estimate is calculated for the expected rate of breaks for each gene based upon gene size and rate of breaks per sample, with a p value obtained based upon (observed-expected)/standard error. A corrected p value of < 0.05 is considered significant. Manual inspection of copy number plots was undertaken to ensure sufficient probe coverage was present at identified loci in order to prioritise the most convincing breakpoints. Those with substantial gaps at either side of the break were excluded.

### Custom oligonucleotide array CGH

We designed two fine-tiling oligonucleotide microarrays to cover the specific amplicons observed at chromosome 2p22.1 and 10q11.21 This was undertaken using the Agilent custom array design tool e-Array (Agilent, Santa Clara, CA, USA; https://earray.chem.agilent.com/), and comprised 700 probes covering 43.56–43.70 Mb on chromosome 10 and 5000 probes covering 39–40 Mb on chromosome 10 with a median probe interval of 200 bp on 2x105K microarray. Due to limited amount of material, DNA was whole genome amplified (WGA) using the GenomePlex® Complete Whole Genome Amplification Kit (Sigma, Gillingham, UK) starting with 10 ng of sample and control DNA, and following the manufacturer’s protocol. WGA DNA was labelled using the Agilent Genomic DNA ULS labelling kit, hybridised as per manufacturer’s instructions, and scanned on the Agilent 2505B Microarray Scanner System. Data has been submitted to ArrayExpress with accession number E-MTAB-2340.

### siRNA knockdown

siRNA was carried out using a Dharmacon SMARTpool™ (Dharmacon, Lafayette, CO, USA) against *MAP4K3* (#003588) with paediatric glioma cells SF188, KNS42, UW479, Res259 and Res186 [16] and a panel of breast carcinoma lines. Cells were plated and transfected 24 hours later with siRNA using Lipofectamine RNAiMax™ (Invitrogen, Paisley, UK) as per manufacturer’s instructions, alongside transfections of siControl. Twenty four hours following transfection, cells were trypsinised and media replenished after 48 hours and 96 hours, with cell viability assessed after seven days using CellTiter-Glo™ Luminescent Cell Viability Assay (Promega, Madison, WI, USA) as per manufacturer’s instructions.

## Results

### Intragenic copy number breakpoints in paediatric HGG

**Figure 1:**
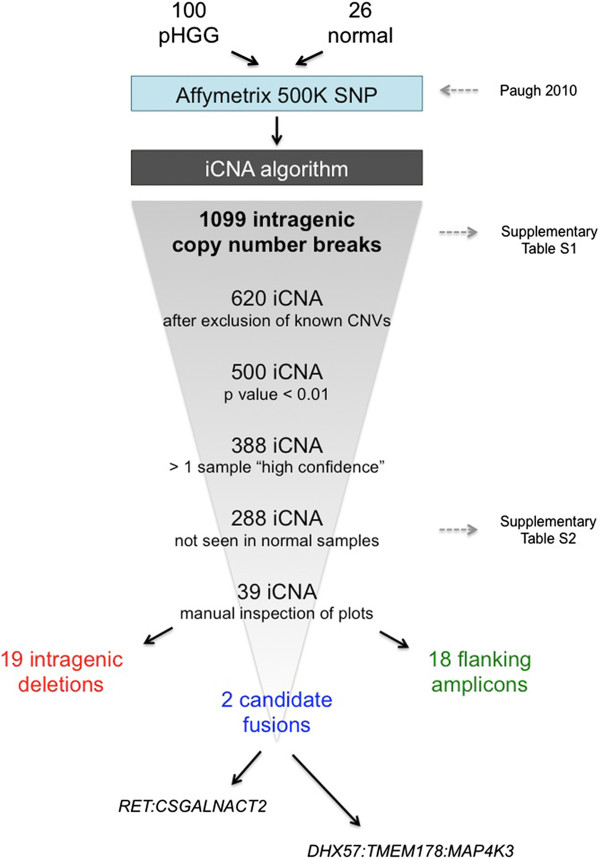
***Schema of iCNA algorithm applied to paediatric high grade glioma.*** 1099 intragenic breakpoints were initially identified in a published series of 100 pHGG. From these, known copy number polymorphisms were excluded, as were these seen in a series of 26 matched normal DNA samples. After filtering for statistical significance and manual inspection of copy number plots, a series of 19 intragenic deletions, 18 amplicons, and 2 candidate fusions were identified.

We have previously carried out DNA copy number profiling of a large series of pHGG samples using Affymetrix 500 K SNP arrays, and reported numerous genes encompassed within areas of focal amplification and deletion [4]. We now applied an algorithm (iCNA [14]) designed to identify copy number breakpoints contained within the sequence of known genes. A full schema of the analytical process is given in Figure 1.

This algorithm was applied to 100 pHGG and 26 matched normal DNA samples, resulting in the identification of 1099 unique DNA copy number breaks contained within gene sequences across all tumour samples (Additional file 1: Table S1). Of these, 479 were found to map to known regions of copy number variations found commonly in the germlines of the general population by cross-referencing the breakpoints with The Centre for Applied Genomics Database of Genomic Variants [17], leaving a total of 620 events.

These were filtered to 500 after excluding those with p values > 0.05, and further reduced to 388 with at least one sample harbouring a given aberration at ‘high confidence’. A further 100 of these were excluded as they were also found in at least one of the normal samples profiled, representing either technical artifacts associated with the array platform used, or low frequency normal copy number polymorphisms. A final list of 288 iCNA is provided in Additional file 2: Table S2.

**Figure 2:**
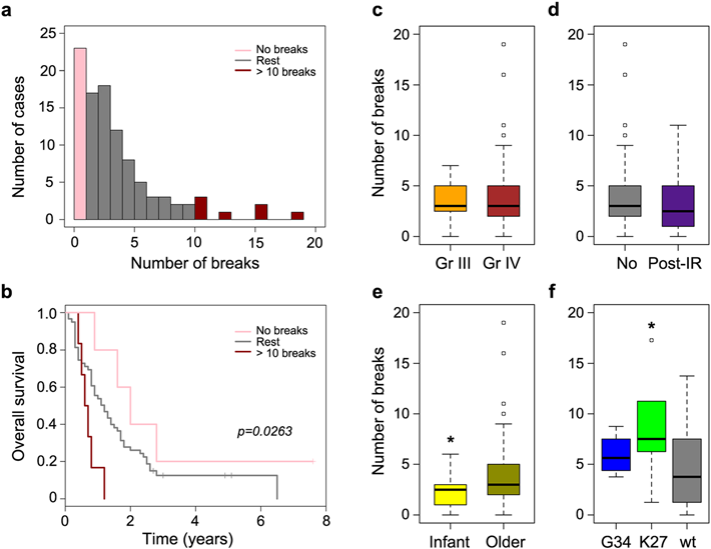
***Clinicopathological correlates of intragenic copy number breaks in paediatric high grade glioma*** **. (a)** Number of intragenic breaks per sample in a series of 100 pHGG. **(b)** Kaplan-Meier plot of overall survival stratified by number of intragenic breaks per sample. Pink: no breaks; Red: more than 10 breaks per sample; Grey: rest. **(c**-**f)** Boxplot of number of intragenic breaks per sample separated by - **(c)** WHO grade. Orange: grade III; Brown: grade IV. **(d)** Previous radiation treatment for an earlier malignancy. Grey: primary pHGG; Purple: post-irradiation; **(e)** Age at diagnosis. Yellow: infant (<3 years old); Dark yellow: older children (>3 years old). **(f)** *H3F3A* status. Blue: G34R/V; Green: K27M; Grey: wild-type.

Most pHGG samples harboured at least one iCNA (median = 3), although seven cases were found to contain none. Several cases were found to contain many more aberrations (maximum = 19), though these were in the minority (Figure 2a). The number of iCNA events per sample was found to be prognostic in this multi-institutional series of cases, with pHGG containing more than 10 iCNA (n = 9) found to have a significantly poorer survival (median = 7.8 months), and those with no iCNA a better survival (median = 24 months) than the rest of the tumours (median = 13.2 months) (p = 0.026, log-rank test) (Figure 2b).

There were no differences in the number of iCNA between grade III (n = 20, median = 3, range 0–7) and grade IV tumours (n = 58, median = 3, range 0–19) (p = 0.456, *t*-test), though the cases with the highest number of iCNA were all grade IV glioblastoma (Figure 2c). Similarly, there were no differences between primary tumours (n = 68, median = 3, range 0–19) and those which arose as secondary malignancies after cranio-spinal irradiation (n = 10, median = 2.5, range 0–11) (p = 0.698, *t*-test) (Figure 2d). Infants (less than 3 years at diagnosis) had significantly fewer iCNA (n = 10, median = 2.5, range 0–6) than older children (n = 68, median = 4, range 0–19) (p = 0.050, *t*-test) (Figure 2e). Tumours with the K27M mutation in the gene encoding the histone variant H3.3, *H3F3A,* harboured significantly more iCNA (n = 5, median = 6, range 1–16) than either G34R/V mutant tumours (n = 4, median = 4.5, range 3–7) or wild-type (n = 14, median = 3, range 0–11) (p = 0.043, ANOVA) (Figure 2f). This was independent of location of tumour, with no differences in number of iCNA between supratentorial GBM (n = 51) and DIPG (n = 7, p = 0.684, *t*-test) (data not shown).

### Intragenic amplifications and deletions

**Figure 3:**
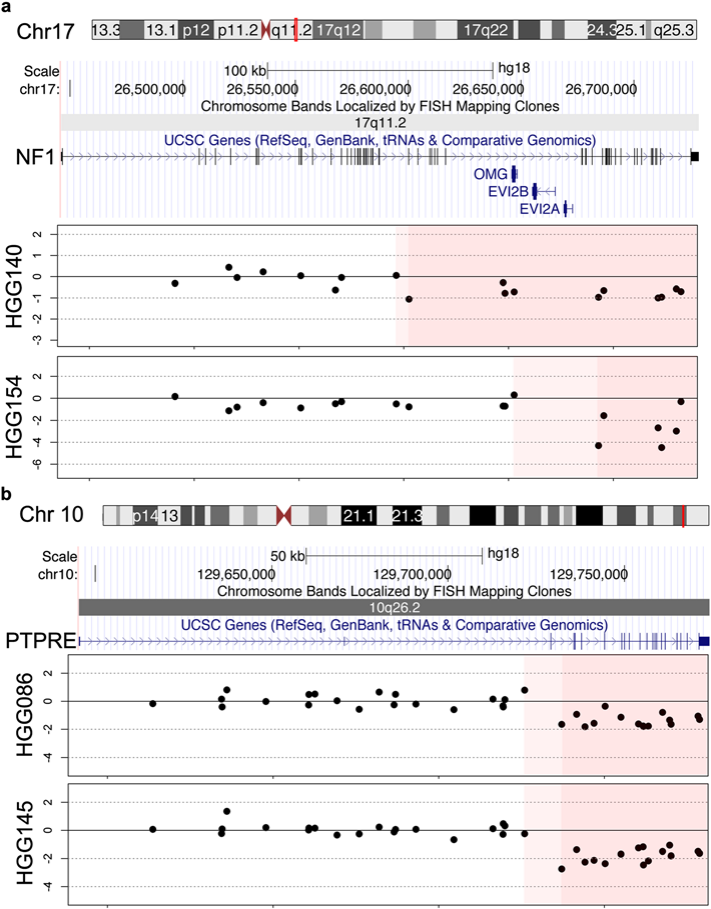
***Intragenic deletions in paediatric high grade glioma.*** **(a)** Recurrent copy number breakpoint within *NF1* on chromosome 17q11.2 in two cases of pHGG. **(b)** Recurrent copy number breakpoint within *PTPRE* on chromosome 10q26.2 in two cases of pHGG. Dark pink: confirmed region of loss; Light pink: region within which breakpoint lies, as defined by the resolution of probes on the array.

The 288 iCNA were further subjected to individual manual inspection of the data plots in order to identify the most robust copy number shifts associated with intragenic breaks. This resulted in a list of 39 unique events in 51 samples (Table 1). The recurrent changes included copy number loss, resulting primarily in either the absence of the 3′ end of a gene or small deletions wholly within the coding sequence. These intragenic deletions included those targeting known tumour suppressors in glioblastoma such as *NF1* (17q11.2, n = 2) (Figure 3a) and *RB1* (13q14.2, n = 1) (Additional file 3: Figure S3), as well as putative novel GBM-associated genes including *FAF1* (1p33, n = 2) and MTAP (9p21.3, n = 2) (Additional file 3: Figure S3). In addition, there were novel deletions in the protein phosphatase *PTPRE* (10q26.2, n = 2) (Figure 3b) and recurrent internal microdeletions in the gene *CSMD3* (CUB and Sushi multiple domains 3) (8q23.3, n = 3), all of which overlapped to result in the loss of exon 4 (Additional file 3: Figure S3).

**Table 1:** **Nominated intragenic copy number aberration candidates**

Candidate.ID	Gene	Chromosome	Sample	Copy number change	Comments
1	FAF1	1	HGG091	Deletion	
	FAF1	1	HGG140	Deletion	
2	CD84	1	HGG088	Amplification	
3	LGALS8	1	HGG070	Deletion	
4	KIDINS220	2	HGG077	Amplification	
5	DHX57	2	HGG063	Amplification	Fusion candidate
6	TMEM178	2	HGG063	Amplification	Fusion candidate
7	WDR49	3	HGG010	Amplification	
8	PDCD10	3	HGG157	Deletion	
9	CHIC2	4	HGG077	Amplification	
10	ITGA1	5	HGG029	Deletion	
11	EPHA7	6	HGG139	Deletion	
12	LANCL2	7	HGG060	Amplification	
13	ECOP	7	HGG060	Amplification	
14	KCND2	7	HGG152	Amplification	
	KCND2	7	HGG162	Amplification	
14	SND1	7	HGG090	Deletion	
15	CSMD3	8	HGG054	Deletion	
	CSMD3	8	HGG140	Deletion	
	CSMD3	8	HGG153	Deletion	
16	SLC24A2	9	HGG151	Deletion	
	SLC24A2	9	HGG011	Deletion	
17	MTAP	9	HGG022	Deletion	
	MTAP	9	HGG007	Deletion	
18	ANKRD26	10	HGG068	Deletion	
19	RET	10	HGG139	Amplification	Fusion candidate
20	CSGALNACT2	10	HGG139	Amplification	Fusion candidate
21	PTPRE	10	HGG086	Deletion	
	PTPRE	10	HGG145	Deletion	
22	RAB6IP1	11	HGG092	Amplification	
23	PSMA1	11	HGG092	Amplification	
24	TMTC1	12	HGG010	Amplification	
	TMTC1	12	HGG068	Amplification	
25	LRRK2	12	HGG068	Amplification	
26	MYO1A	12	HGG029	Amplification	
27	XRCC6BP1	12	HGG029	Amplification	
28	OSBPL8	12	HGG065	Amplification	
	OSBPL8	12	HGG162	Amplification	
29	RB1	13	HGG154	Deletion	
30	PCDH17	13	HGG059	Deletion	
31	CD276	15	HGG006	Deletion	
32	MEF2A	15	HGG011	Deletion	
33	DNAH2	17	HGG143	Amplification	
34	NF1	17	HGG154	Deletion	
	NF1	17	HGG140	Deletion	
35	BRIP1	17	HGG077	Deletion	
36	KCNB1	20	HGG139	Amplification	
37	SYN3	22	HGG017	Deletion	
	SYN3	22	HGG146	Deletion	
38	TIMP3	22	HGG146	Deletion	
39	PHF21B	22	HGG072	Deletion	

39 unique intragenic breakpoints found within 51 cases of paediatric high grade glioma. Direction of copy number shift (gain/loss) is reported, as well as candidate fusion events.

**Figure 4:**
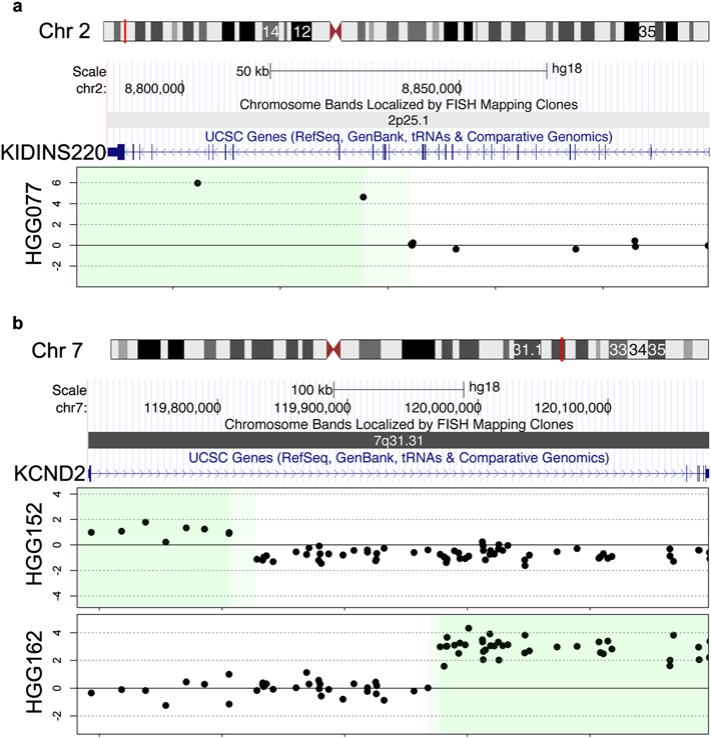
***Intragenic amplifications in paediatric high grade glioma.*** **(a)** Copy number breakpoint within *KIDINS200* on chromosome 2p25.1, flanking the *MYCN* amplicon in a case of pHGG. **(b)** Recurrent copy number breakpoint within *KCND2* on chromosome 7q31.31 in two cases of pHGG, in both cases part of the *MET* amplicon, though targeting either the 5′ or 3′ end of the gene. Dark green: confirmed region of gain; Light green: region within which breakpoint lies, as defined by the resolution of probes on the array.

Copy number gains within gene coding regions tended to be associated with regions flanking known oncogenic amplicons. These included amplification of the *MYCN* locus at chromosome 2p24.3, which in case HGG077 breaks within the coding region of the kinase D-interacting substrate *KIDINS220* (Figure 4a); amplification of *PDGFRA* at 4q12, harbouring an iCNA in *CHIC2* in the same case (though only covered by two probes); and recurrent breakpoints in the gene encoding the potassium voltage-gated channel *KCND2* at 7q31.31 in association with amplification of *MET*, though curiously this targeted either 5′ or 3′ ends in two different cases (Figure 4b). Similarly, common amplification events encompassing *EGFR* (7p12) and *CDK4* (12q14) had intragenic breakpoints at both ends in cases HGG060 (*LANCL2* and *ECOP*) and HGG029 (*MYO1A* and *XRCC6BP1*), respectively (Additional file 4: Figure S4).

### Identification of novel fusion genes

For the most part, iCNA events resulted in an imbalance of certain regions of coding genes in isolation, with the predicted consequence a disruption of full-length gene expression. For certain events however, a 5′ end of one gene was found amplified at a similar copy number to a 3′ end of a second gene within the same case. We reasoned that such instances may represent candidate fusion genes, and we identified two such examples in our cohort.

The first was at chromosome 10q11.21 and reflected a single amplicon, breaking within the genes *RET* and *CSGALNACT2* such that we propose a hypothetical fusion gene encompassing the 5′ regulatory regions of *CSGALNACT2* and the 3′ kinase domain of *RET*. In order to determine the precise breakpoints to allow validation of this novel fusion, we designed custom oligonucleotide arrays spanning the amplicon in order to carry out high-resolution array CGH on the reference case HGG139, a relapse sample of glioblastoma in which this genomic event was not present in the primary tumour. Although the breakpoint for *CSGALNACT2* was identified within intron 2, leaving the catalytic domains intact, the breakpoint within *RET* could not be accurately determined to closer than 10 kb between introns 1 and 2 (Additional file 5: Figure S5). As material was limited for this case, we were unable to confirm the precise nature of the putative *CSGALNACT2:RET* fusion by PCR-based techniques.

**Figure 5:**
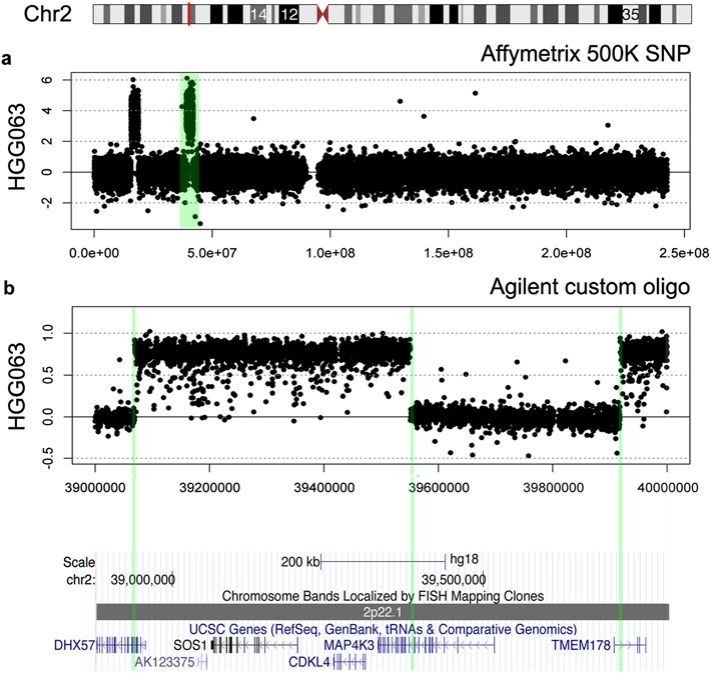
***Identification of a novel complex fusion DHX57:TMEM178:MAP4K3.*** **(a)** Affymetrix 500 K SNP array of chromosome 2, highlighting two amplicons, the most telomeric encompassing *MYCN*, the more centromeric as 2p22.1 involving intragenic breakpoints in *DHX57* and *TMEM178* (green). **(b)** Custom oligonucleotide array of the 2p22.1 amplicon, revealing two amplified structures and three intragenic breakpoints, in *DHX57, MAP4K3* and *TMEM178* (green).

**Figure 6:**
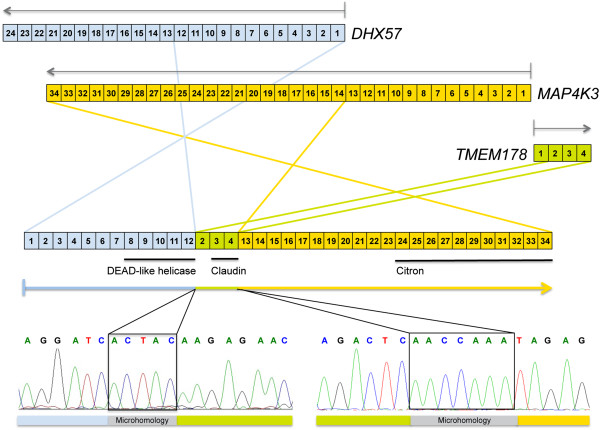
**Cartoon depicting the structure of the** ***DHX57:TMEM178:MAP4K3*** **fusion.** A complex microhomology-mediated rearrangement of exons 1–12 of *DHX57* (blue), exons 2–4 of *TMEM178* (green) and exons 13–34 of *MAP4K3* (orange) was confirmed by direct sequencing. Regions of microhomology are highlighted in grey.

The second fusion candidate was located at an amplified region of chromosome 2p22.1 in case HGG063, an anaplastic astrocytoma. At Affymetrix 500 K SNP resolution, this appeared to be a single amplicon with breaks within the coding regions of the RNA helicase *DHX57* and the transmembrane protein *TMEM178* (Figure 5a). Applying the same approach as above, using custom-designed oligonucleotide arrays for high-resolution array CGH revealed two amplicons within this structure, with further intragenic breakpoints within the mitogen-activated protein kinase *MAP4K3* (Figure 5b). Designing PCR primers to amplify across the highly specific breakpoints confirmed the presence of the fusion, which was further validated by direct sequencing (Figure 6).

The resultant fusion gene, *DHX57:TMEM178:MAP4K3*, is a complex three gene fusion formed from a series of intragenic breaks, amplifications and inversions to produce a sequence comprising exons 1–12 of *DHX57*, exons 2–4 of *TMEM178* and exons 13–34 of *MAP4K3,* associated with regions of microhomology (Figure 6). This would produce a protein with the zinger finger and DEAD-like helicase domains of DHX57, the claudin family transmembrane domains of TMEM178 and the citron domain of MAP4K3. *S*elective knockdown of MAP4K3 by siRNA leads to a significant reduction in cell viability in five paediatric glioma cell lines as assayed by CellTiter Glo, an effect not seen in 18/20 breast cancer cells (p = 0.0017, pHGG *vs* breast cancer, *t*-test) (Additional file 6: Figure S6).

## Discussion

Comprehensive copy number profiling of adult and paediatric high grade gliomas was among the first data to demonstrate the biological differences between these similar-looking histological malignancies [18]. In this context, the focus has been on large-scale genomic copy number changes. A more refined analysis of copy number and exon-level expression data has identified new insights into genomic architecture and novel fusion proteins in adult glioblastoma [12, 13]. Here we leverage a large dataset we have previously generated [4] in the paediatric disease to carry out a scan of intragenic breakpoints, leading to the identification of novel gene disruptions and candidate gene fusions.

The presence of intragenic copy number aberrations was confirmed in the vast majority of pHGG cases, and was itself prognostic, with an absence of iCNAs conferring a longer overall survival in paediatric patients. This was associated with the infant age group, known to have a better clinical outcome than older children [19], and further highlights the biological distinctiveness of this age group. By contrast, the presence of large numbers of intragenic breaks conferred a shorter survival time, but was not a result of the grade of the tumour, nor associated with a second malignancy due to radiation treatment for an earlier cancer. We had previously reported an association of post-irradiated HGG with *PDGFRA* amplification and chromosome 1q gain [4], so it appears these are relatively selective radiation-induced changes, rather than reflecting a generalised genomic instability in secondary tumours from these patients. Importantly, we identified an increased number of iCNA in tumours harbouring an *H3F3A* K27M mutation, regardless of anatomical location. This is a group of thalamic and pontine HGG associated with a particularly dismal prognosis [18], for whom understanding the mechanisms of genomic instability and the identification of novel gene disruptions is of considerable interest.

The majority of intragenic breakpoints we identified were associated with gene disruption. This includes deletions of known tumour suppressors such as *RB1* and *NF1*, but also more novel glioblastoma associated genes. *FAF1* and *MTAP* were both recurrently targeted by intragenic deletion events in pHGG. These genes are localised close to known cyclin-dependent kinase inhibitors and tumour suppressors *CDKN1C* and *CDKN2A/B*, respectively, but both *FAF1* and *MTAP* have recently been proposed to harbour tumour suppressor activity in their own right. *FAF1* is associated with a FAS-mediated apoptosis response and restoration of the FAF1 protein in adult glioma cell lines significantly increases cell death [20], whilst in MTAP-deficient cells, methylthioadenosine, generated during polyamine biosynthesis, is not cleaved and the salvage pathway for adenine and methionine is absent [21]. It seems that such mechanisms are also likely in a subset of paediatric tumours.

Of note we identified novel deletions in the protein phosphatase epsilon, *PTPRE*. This has not been reported previously, although there are several reports of the tumour suppressive capacity of the related *PTPRD*[22, 23]. This gene also appears targeted by intragenic deletions, and human astrocytes lacking PTPRD exhibited increased growth, as it is thought the protein usually functions to dephosphorylate the oncoprotein STAT3 [23]. The wholly intragenic microdeletions observed in *CSMD3* in four cases may represent another novel mechanism of gene disruption. *CSMD3* encodes a gene with multiple CUB and Sushi domains whose function is poorly understood. Recently, *CSMD3* was identified as the second most frequently mutated gene (next to *TP53*) in lung cancer, where it was demonstrated that loss of CSMD3 results in increased proliferation of airway epithelial cells [24].

Gene disruption may also play a significant functional role when known gain-of-function oncogenes are amplified. We report numerous intragenic breakpoints which may have been overlooked in the context of identifying the ‘driver’ event within a common amplicon, but which may themselves be tumorigenic. These include disruptions of *KIDINS220*, a functional mediator of multiple receptor signalling pathways and essential for cortical development [25, 26]; *CHIC2,* frequently deleted/rearranged in myeloid malignancies [27]; and *KCND2*, encoding a potassium voltage-gated channel, which is expressed in both neuronal and glial cells and has been shown to regulate ERK signalling in ganglioglioma [28]. All of these gene disruptions represent novel avenues for understanding the underlying biology of pHGG.

Of most interest was the use of the iCNA algorithm to identify potential novel fusion genes, as was demonstrated in adult glioblastoma with the identification of the *KDR:PDGFRA* fusion [10], which we also found in a case of pHGG [11]. Our analysis nominated two potential candidates – the first we were unable to conclusively validate, *CSGALNACT2:RET*. Such a putative fusion would retain the kinase domain of the RET oncoprotein, but would lose the autoregulatory portion of the protein, instead fusing it to the N terminal of chondroitin sulfate N-acetyl-galactosaminyltransferase 2. Although a precise cancer-related function has not been ascribed to the latter enzyme, it is though to play an important role in morphogenesis in zebrafish models [29, 30]. Whilst not validated, oncogenic *RET* rearrangements and fusions are common in thyroid and lung cancer [31, 32], and the presence of infrequent activating fusions in HGG do not seem unlikely.

We were able to validate a novel complex fusion involving three genes with intragenic breakpoints and amplification/rearrangement on chromosome 2p22.1. The resulting fusion gene, *DHX57:TMEM178:MAP4K3* encompasses key regulatory domains from all three proteins, though a specific function is hard to predict. The helicase properties of the DHX57 component may be a candidate for oncogenicity, with numerous other DEAD-box helicases appearing to play a role in regulation of DNA repair, apoptosis and drug sensitivity [33]. *MAP4K3* has been associated with several malignancies in both an oncogenic and tumour suppressor capacity [34, 35]. In particular, one function that has been ascribed includes activation of mTOR signalling via the TORC1 complex [36], a pathway commonly activated by diverse mechanisms in pHGG [18].

In the context of pHGG, although the kinase domain is not retained in the fusion, MAP4K3 plays some functional role as selective knockdown by siRNA leads to a significant and selective reduction in cell viability in paediatric glioma cell lines. Thus we hypothesise that the *DHX57:TMEM178:MAP4K3* is activating as disruption of the protein would otherwise seem incompatible with tumour cell growth and proliferation.

## Conclusion

In summary these data represent a key addition to our understanding of the genomic alterations driving pHGG and provide novel avenues for developing sorely-needed novel therapeutic strategies for children with these otherwise incurable tumours.

## Electronic supplementary material

Additional file 1: Table S1: Initial output from iCNA algorithm detailing 1099 intragenic copy number breakpoints in 100 cases of paediatric high grade glioma. (XLSX 124 KB)

Additional file 2: Table S2: Final output from iCNA algorithm detailing 288 filtered intragenic copy number breakpoints in 100 cases of paediatric high grade glioma. (XLSX 72 KB)

Additional file 3: Figure S3: *Intragenic deletions in paediatric high grade glioma.* (a) Recurrent copy number breakpoint within *FAF1* on chromosome 1p33 in two cases of pHGG. (b) Recurrent copy number breakpoint within *MTAP* on chromosome 9p21.3 in two cases of pHGG. (c) Copy number breakpoint within *RB1* on chromosome 13q14.2 in a case of pHGG. (b) Recurrent copy number breakpoint within *CSMD3* on chromosome 8q23.3 in three cases of pHGG. Dark pink: confirmed region of loss; Light pink: region within which breakpoint lies, as defined by the resolution of probes on the array. (TIFF 838 KB)

Additional file 4: Figure S4: *Intragenic amplifications in paediatric high grade glioma.* (a) Copy number breakpoints within *LANCL2* and *ECOP* on chromosome 7p11.2, flanking the *EGFR* amplicon in a case of pHGG. (b) Recurrent copy number breakpoints within *MYO1A1* and *XRCC6BP1* on chromosome 12q13.3 and 12q14.1, flanking the *CDK4* amplicon in a case of pHGG. Dark green: confirmed region of gain; Light green: region within which breakpoint lies, as defined by the resolution of probes on the array. (TIFF 624 KB)

Additional file 5: Figure S5: *Identification of a novel candidate fusion CSGALNACT2:RET.* (a) Affymetrix 500 K SNP array of chromosome 10, highlighting an amplicon at 10q11.21 (green). (b) Custom oligonucleotide array of the 10q11.21 amplicon, revealing a clear breakpoint within *CSGALNACT2* (green), but a less clear boundary within *RET* (grey). (TIFF 657 KB)

Additional file 6: Figure S6: *siRNA knockdown of MAP4K3 in paediatric glioma and breast carcinoma cells.* Paediatric glioma cells (green) were highly sensitive to knockdown of MAP4K3, with 5/5 cells showing significant effects on cell viability. By contrast, only 2/20 breast cancer cells (blue) showed a similar dependency on MAP4K3 expression for cell viability. The screen was carried out in three independent experiments and was highly reproducible for all cell lines, with R^2^ values ranging from 0.68-0.94 (breast) and 0.78-0.92 (glioma). The different sensitivity of glioma cells to MAP4K3 knockdown as compared to breast carcinoma cells was statistically significant (p = 0.0017, pHGG *vs* breast cancer, *t*-test). (TIFF 194 KB)
