# Supplementary figures and images for: The prognostic role of intragenic copy number breakpoints and identification of novel fusion genes in paediatric high grade glioma
Source: Acta Neuropathol Commun. 2014 Feb 18;2:23. doi: 10.1186/2051-5960-2-23 (PMC3938307; doi:10.1186/2051-5960-2-23)

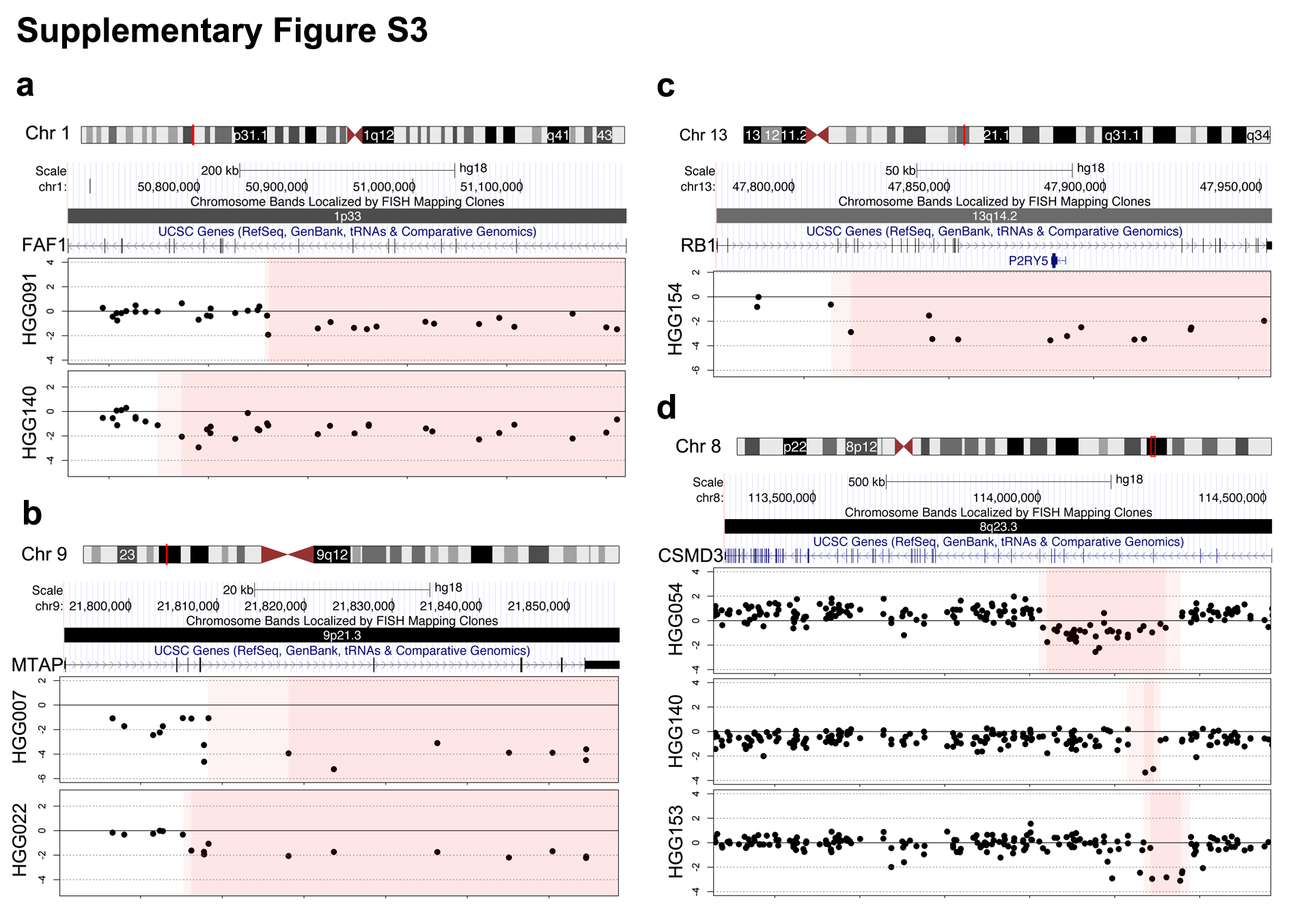

Supplement: Supplementary file 3 — Additional file 3: Figure S3: Intragenic deletions in paediatric high grade glioma. (a) Recurrent copy number breakpoint within FAF1 on chromosome 1p33 in two cases of pHGG. (b) Recurrent copy number breakpoint within MTAP on chromosome 9p21.3 in two cases of pHGG. (c) Copy number breakpoint within RB1 on chromosome 13q14.2 in a case of pHGG. (b) Recurrent copy number breakpoint within CSMD3 on chromosome 8q23.3 in three cases of pHGG. Dark pink: confirmed region of loss; Light pink: region within which breakpoint lies, as defined by the resolution of probes on the array. (TIFF 838 KB) [file 40478_2013_98_MOESM3_ESM.tiff]

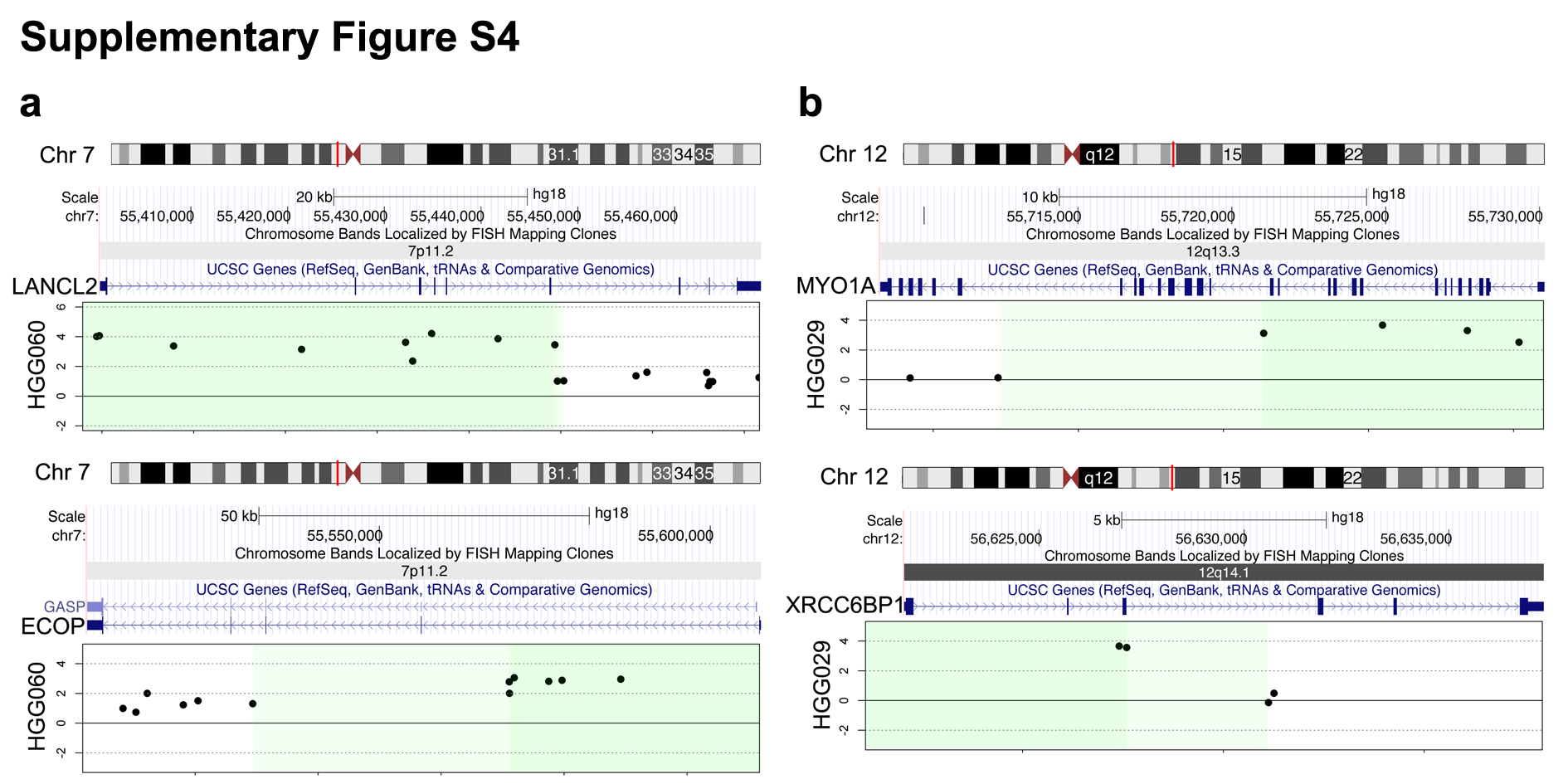

Supplement: Supplementary file 4 — Additional file 4: Figure S4: Intragenic amplifications in paediatric high grade glioma. (a) Copy number breakpoints within LANCL2 and ECOP on chromosome 7p11.2, flanking the EGFR amplicon in a case of pHGG. (b) Recurrent copy number breakpoints within MYO1A1 and XRCC6BP1 on chromosome 12q13.3 and 12q14.1, flanking the CDK4 amplicon in a case of pHGG. Dark green: confirmed region of gain; Light green: region within which breakpoint lies, as defined by the resolution of probes on the array. (TIFF 624 KB) [file 40478_2013_98_MOESM4_ESM.tiff]

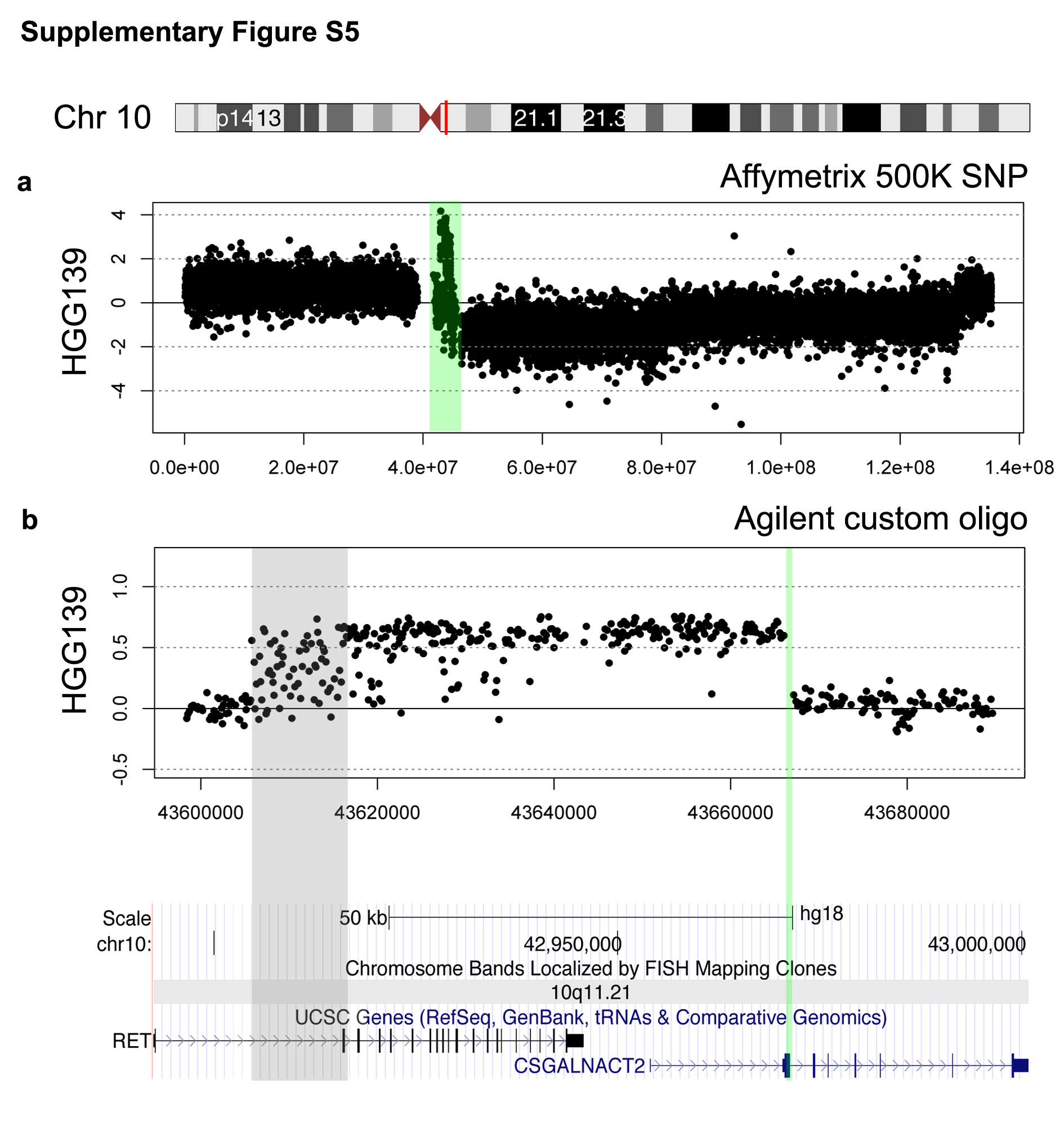

Supplement: Supplementary file 5 — Additional file 5: Figure S5: Identification of a novel candidate fusion CSGALNACT2:RET. (a) Affymetrix 500 K SNP array of chromosome 10, highlighting an amplicon at 10q11.21 (green). (b) Custom oligonucleotide array of the 10q11.21 amplicon, revealing a clear breakpoint within CSGALNACT2 (green), but a less clear boundary within RET (grey). (TIFF 657 KB) [file 40478_2013_98_MOESM5_ESM.tiff]

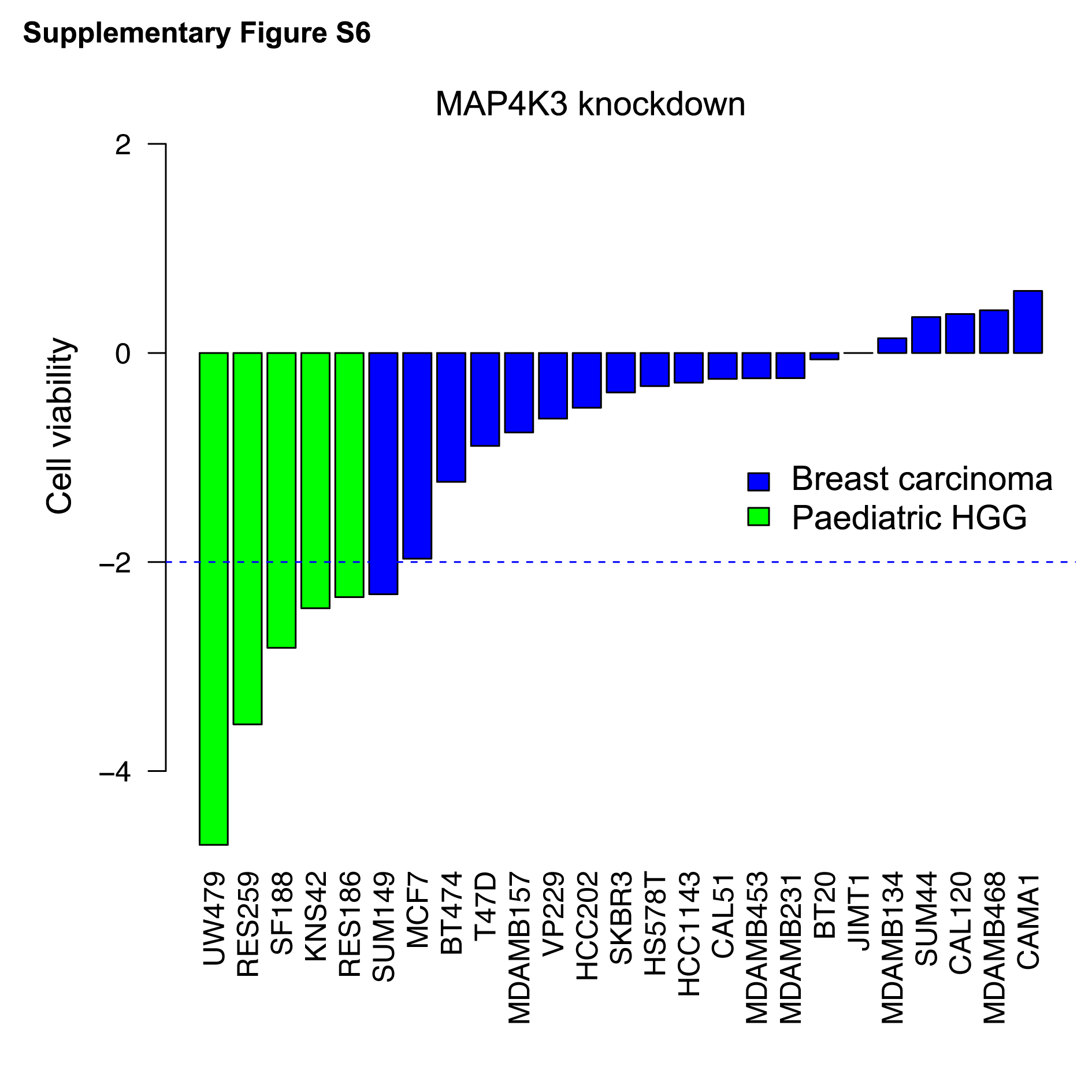

Supplement: Supplementary file 6 — Additional file 6: Figure S6: siRNA knockdown of MAP4K3 in paediatric glioma and breast carcinoma cells. Paediatric glioma cells (green) were highly sensitive to knockdown of MAP4K3, with 5/5 cells showing significant effects on cell viability. By contrast, only 2/20 breast cancer cells (blue) showed a similar dependency on MAP4K3 expression for cell viability. The screen was carried out in three independent experiments and was highly reproducible for all cell lines, with R2 values ranging from 0.68-0.94 (breast) and 0.78-0.92 (glioma). The different sensitivity of glioma cells to MAP4K3 knockdown as compared to breast carcinoma cells was statistically significant (p = 0.0017, pHGG vs breast cancer, t-test). (TIFF 194 KB) [file 40478_2013_98_MOESM6_ESM.tiff]
